# Elevating Elderly Cancer Care: A Systematic Review of Advanced Practice Nursing's Role in Senior Oncology Patients' Quality of Life

**DOI:** 10.1155/2024/6698804

**Published:** 2024-10-10

**Authors:** Cristian-David Useche-Guerrero, María-de-los-Ángeles Merino-Godoy, Eva-María Barroso-Márquez, Emilia Isabel Martins Teixeira da Costa, Rafaela Camacho Bejarano, Francisco-Javier Gago-Valiente

**Affiliations:** ^1^ Nursing Department Faculty of Nursing University of Huelva, Huelva 21007, Spain; ^2^ Nursing Department Health School University of Algarve, Faro 8000, Portugal; ^3^ Health Sciences Research Unit: Nursing Nursing School of Coimbra, Coimbra 3000, Portugal; ^4^ Center for Research Contemporary Thought and Innovation for Social Development (COIDESO) University of Huelva, Huelva 21007, Spain

## Abstract

**Background:**

According to projections based on current trends, it can be anticipated that from 2024 onward, approximately 70% of all cancer cases will be diagnosed in individuals 65 years and older. Given this complex intersection between population ageing and cancer incidence, it is of great importance to address this issue from a comprehensive care perspective. Here comes the importance of advanced practice nurse into play. However, this figure is still not sufficiently valued in many countries. Its roles are also not clearly defined at the international level. For this reason, a systematic review of the scientific literature was carried out to analyze the impact of advanced practice nurse on the quality of life of older adults with cancer.

**Methods:**

Searches were carried out in PubMed, Web of Science (WoS), Scopus, CINAHL, LILACS, and ScienceDirect databases. They were limited to studies conducted in the last 7 years. Only open-access articles were selected. To analyze the chosen articles and assess their quality, the criteria of the PRISMA and CASPe statements were applied. All authors participated in both the selection of the articles and their analysis.

**Results:**

Of the initial 58 articles selected, a total of 10 articles were finally included, as they met the eligibility criteria established after further analysis. The results show a positive relationship between the advanced practice nurse intervention and quality of life in older adults with cancer.

**Conclusions:**

Advanced practice nurse (APN) plays a key role in the care of older adults with cancer, significantly improving their quality of life and contributing to the comprehensive care of these patients. The findings evidenced in this work support the integration of APNs in cancer care teams to improve patient experience and overall well-being.

## 1. Background

A major challenge is being grappled with modern society: the intertwined issues of population growth and rising life expectancy. Life has been expanded by advances in healthcare, but a complex scenario has been created by declining fertility rates. From 1950 to 2015, the population over 60 quadrupled, and it is projected to be 2.1 billion by 2050 [1]. Public health worldwide is being transformed by demographic changes due to evolving factors, with significant impacts on both health and social aspects [2].

An important concern in this context is the growing prevalence of chronic diseases related to age, including cardiovascular problems, degenerative conditions, dementia, and notably, cancer [1]. From the analysis of the most current statistics, it can be seen that around 20 million new cases of cancer were reported worldwide in 2020. Among these, breast, lung, colon, prostate, and stomach cancers are noted as standing out, being currently recognized as some of the diseases with the highest incidence rates on the international scene [3]. By 2024 and beyond, around 70% of cancer cases are expected to be diagnosed in people 65 years and older, driven by ageing-related biological changes and a higher likelihood of comorbidities. This poses a significant risk to the quality of life for older individuals [4, 5].

To tackle the challenges of ageing and increased cancer rates, a holistic strategy is essential. Nursing plays a crucial role by adopting a comprehensive perspective, considering individual differences, and tailoring personalized solutions for better elderly life quality [6].

Quality of life in cancer patients is a key concept in nursing, encompassing general well-being in various dimensions. Its understanding has evolved since its origin in the post-World War II in the United States [7]. Originally, quality of life in cancer patients was assessed through objective measures such as survival and physical function. However, psychologists found that these could not fully explain variations. Subjective interpretations, such as happiness and life satisfaction, became crucial. These subjective factors often have a more significant impact on the quality of life of patients than objective factors alone [7].

In this context, the concept of health-related quality of life (HRQoL) emerged to capture the interaction between health and quality of life in a more holistic way [7]. To measure the HRQoL in cancer patients effectively, it is essential to consider multiple components such as functional, cognitive, emotional, spiritual, social, and economic aspects. Assessing specific problems and symptoms, such as pain and fatigue, is crucial. This comprehensive approach not only aids in clinical decision-making but also identifies vulnerable patient groups that need personalized care. It helps to evaluate the impact of treatment on quality of life, tolerability, and adherence. Nursing plays a key role in providing complete care and improving the overall quality of life for cancer patients [4].

In January 2018, the Andalusia Health Quality Agency (Spain) presented a professional competency manual for advanced practice nurses (APNs) in cancer care that includes competence in complex care coordination, such as in the case of the elderly with cancer [8, 9]. In Spain, there is a distinction between generalist and specialist nurses. However, with the increasing demand for health services and the need for highly specialized care, the new role of APNs has arisen. This model draws inspiration from Anglo-Saxon countries such as the United Kingdom, Canada, Australia, and New Zealand, adapting to the evolving complexity of health systems [5]. In the international context, advanced practice nurse is increasingly gaining momentum in different countries. However, there is a common denominator which is the need to provide clarity about this role in the provision of health services [10].

The concept of the APN originated in the United States during the 1960s and was later adopted in other countries, including the United Kingdom, Canada, and Australia. Following the National Cancer Act of 1971, which established a comprehensive framework for cancer prevention, diagnosis, and treatment in the U.S., APNs initially focused on cancer management and research. As treatments grew more complex, the need for improved coordination across cancer care facets became evident, leading to stronger interdisciplinary collaboration and integrated care. The United Kingdom's Calman–Hine's report recommended specialized multidisciplinary teams and oncology care networks, reflecting these needs. Similarly, in other countries such as Portugal, this role is fulfilled by specialized nurses who, although they may not carry the same title, perform comparable functions in providing expert care and coordinating healthcare services in their areas of specialization [8].

An APN is a highly skilled nursing professional who has obtained advanced educational credentials and clinical training beyond the basic nursing education and licensing required of a registered nurse (RN). APNs are prepared through a postgraduate degree, such as, a master's or doctoral program, which enables them to provide a higher level of care and take on roles that include direct patient care, consultation, education, research, and administration [8].

ICN emphasizes that APNs should conduct direct healthcare practices within their focus population. These nurses can play a crucial role in assessing and diagnosing the needs of senior oncology patients using theories such as the theory of unpleasant symptoms and the symptom management theory. Their expertise helps identify clusters of symptoms and assess their impact on patient's quality of life and functionality [11].

APNs play a multifaceted role in the care of elderly patients diagnosed with cancer, functioning across various capacities that directly impact patient outcomes. As consultants, they provide crucial guidance to healthcare professionals, patients, and families, ensuring personalized and understandable care plans. Their educational responsibilities are profound, with a focus on disseminating advanced nursing practices to clinical nurses in primary care settings and sociohealthcare residences, areas critical to the elderly population. APNs also engage in research, contributing to the development of innovative, evidence-based approaches that enhance cancer care. Furthermore, through transformational leadership, APNs implement significant changes in healthcare practices, advocating for improvements that optimize the treatment and recovery processes for senior oncology patients. These roles exemplify the APNs' integral contribution to a holistic healthcare approach, emphasizing their importance in both direct patient care and broader healthcare improvements [8].

Although the APN role is recognized globally, there is a scarcity of studies examining its impact on the quality of life of senior oncology patients. Updated information is crucial, especially postpandemic, to understand the quality of service perceived by the patient and academic variables related to proper training in this role. Older patients are a heterogeneous population ranging from those who are frail and dependent to those who are extremely active. Therefore, it is necessary to provide adequate geriatric care. Currently, there is not enough evidence on whether nursing staff apply adequate and comprehensive care to senior oncology patients. APNs could have a relevant role in this comprehensive care, which is why it is important to know the experiences that already exist with these professionals [12].

Therefore, the objective of this research was to conduct a systematic review of the scientific literature to identify research studies that analyze the impact of APNs on the quality of life of older adults with cancer. This study further examines evidence of the effectiveness of advanced practice nurse roles in meeting the healthcare needs specifically the quality of life of senior oncology patients.

## 2. Materials and Methods

In this study, a systematic review of the scientific literature was carried out in which studies analyzed the relationship between APN interventions and the quality of life of older adults with cancer. PRISMA statement criteria were applied [13] for systematic reviews by comprehensively analyzing the selected articles. The study was carried out according to the guidelines of the Declaration of Helsinki. This research is registered in PROSPERO (International Prospective Register of Systematic Reviews) with the registration number 488680.

The PICO framework is a structured approach that helps in formulating precise clinical research questions. In the proposed study, the focus is on senior oncology patients (population), investigating the effects of advanced practice nurse care (intervention) in comparison to standard nursing care (comparison). The main goal is to determine the benefits of such specialized nursing interventions in terms of improved health outcomes (outcome). Accordingly, the research question formulated is as follows: “What are the benefits of advanced practice nurse care in improving health outcomes compared to standard nursing care among senior oncology patients?” This question aims to capture the specific impacts of advanced practice nurse on the care quality and health results in this vulnerable group, thereby guiding potential improvements in clinical practices.

### 2.1. Selection Criteria

The systematic review was carried out between February and March 2023 in PubMed, Web of Science (WoS), SCOPUS, CINAHL, LILACS, and ScienceDirect databases. The investigation focused on literature published within the past seven years, specifically from January 1, 2016, to March 31, 2023, and exclusively included open-access documents. Our aim was to derive recent and well-supported findings from a range of documentary materials, leading to the decision to incorporate both research studies and reviews, whether systematic or bibliographic in nature.

### 2.2. Search Strategy

The search strategy was approached by selecting the following search criteria: for PubMed ((“Advanced Practice Nurse” [Mesh]) AND “Aged” [Mesh]) AND “Neoplasms” [Mesh]; ((“Advanced Practice Nurse” [Mesh]) AND “Neoplasms” [Mesh]) AND “Quality of Life” [Mesh]; for WOS ((ALL = (advanced practice nurse)) AND ALL = (neoplasms)) AND ALL = (quality of life); ((TS = (advanced practice nurse)) AND TS = (neoplasms)) AND TS = (quality of life); ((ALL = (advanced practice nurse)) AND ALL = (aged)) AND ALL=(neoplasms); for SCOPUS (TITLE-ABS-KEY (“advanced practice nurse”) AND TITLE-ABS-KEY (neoplasms) AND TITLE-ABS-KEY (aged)) AND PUBYEAR >2016 AND PUBYEAR <2023; (TITLE-ABS-KEY (“advanced practice nurse”) AND TITLE-ABS-KEY (neoplasms) AND TITLE-ABS-KEY (“quality of life”)); for CINAHL advanced practice nurse AND quality of life AND older adult AND cancer; advanced practice nurse AND neoplasms AND aged AND quality of life; for LILACS (advanced practice nurse) AND (cancer) AND (older adult) AND (quality of life); (advanced practice nurse) AND (aged) AND (neoplasms) AND (quality of life); for ScienceDirect advanced practice nurse AND quality of life AND cancer AND older adult.

### 2.3. Inclusion and Exclusion Criteria

The following inclusion criteria were used: (a) studies in which APN was analyzed, (b) consideration of quality of life and cancer variables, (c) studies in which the sample was an adult population, and (d) articles published in scientific journals. All articles included in this review had to meet the four criteria detailed above.

Exclusions from this study encompassed various document types, such as editor letters, commentaries, opinions, perspectives, guidelines, standards, and case series. To ensure the reliability and accuracy of our process, three authors (C.U.-G., F.-J.G.-V., and E.C.) independently assessed the relevance of the selected articles to the study's objectives and adherence to the inclusion criteria. When the title, abstract, and keywords of the article were in doubt for inclusion, two other authors were included (R.C.-B.; M.-d.-l.-A.M.-G. and E.-M.B.-M.) to arbitrate the decision on their inclusion or exclusion.

**Figure 1:**
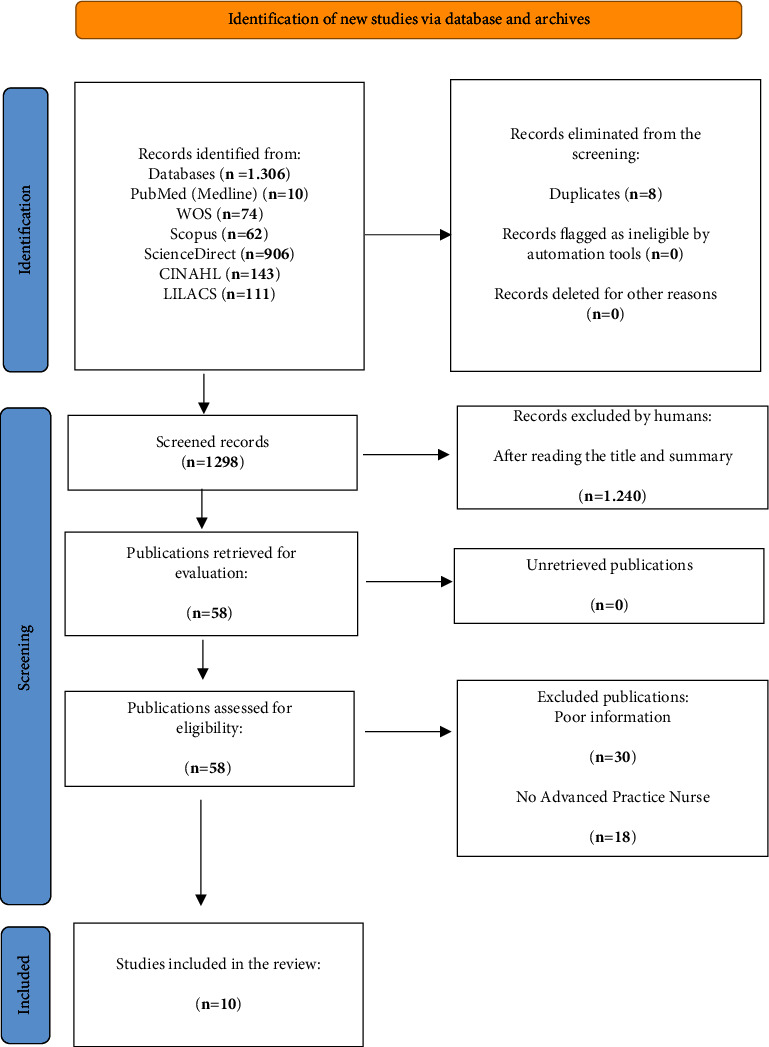
Flowchart of the systematic review process according to the statements of the PRISMA protocol.

The process of identifying and choosing articles, including those that were ultimately included or excluded, as well as the rationale behind their exclusion during the screening and selection stages, is depicted in the flowchart in Figure 1. This representation aligns with the Preferred Reporting Items for Systematic Reviews and Meta-Analyses (PRISMA) guidelines, which are aimed at enhancing the thoroughness in the reporting of systematic reviews and meta-analyses [14].

### 2.4. Data Extraction

The data extraction process was carried out through extensive trials and postsearch proceedings. This started by reviewing, primarily and meticulously, the title, abstract, method, results, and conclusions of each article. Data were extracted as found in their respective studies at the time of review and were inserted into Table 1

In this systematic review, the selection of variables was guided by the PICOS framework [25], encompassing participants, interventions, comparisons, outcomes, and study design. With this strategy, it was possible to delimit the inclusion criteria and, based on them, carry out a qualitative analysis of the results. In addition, the research incorporated other pertinent variables such as the authors, year of publication, country, reference article, study objectives, participant details, variables measured, and the scales used. With data extraction, a document was created with a set of data and was hosted in the Arias Montano Institutional Repository [26].

### 2.5. Presentation of the Results: Adherence to Quality Initiative (PRISMA)

The results of the primary studies, obtained through a systematic and reproducible methodology, were presented qualitatively and quantitatively (Figure 1).

### 2.6. Quality Evaluation

In selecting articles for this review, we conducted a quality analysis using the criteria of the EPHPP tool [27]. This tool assigns an overall quality rating to each study based on the assessment of six key components. Studies are rated as “strong” if they have no weak components and at least four strong ones. Those with fewer than four strong components and one weak component are deemed as “moderate.” Studies receiving two or more weak component ratings are categorized as “weak” [27].

The findings of this analysis are shown in Table 2. Of the various articles analyzed, 14% had a strong overall score [23], 57% a moderate overall score [15, 16, 20, 21], and 29% a weak overall score [17, 18].

However, although the percentage of strong scores was the lowest proportion, all papers had strong internal components compared to the percentage of participants who made it to the end of the intervention. In addition, most studies showed strength in terms of the instruments used for data collection and the risk of bias [15, 16, 20, 23]. These internal components with a strong score are relevant and can be prioritized with respect to others, as they are more closely related to the objective of the study in this systematic review. Therefore, although the presence of other internal components with weak or moderate score is evident, since the most relevant internal components were strong, all these studies were included in this research. Another aspect to highlight is that the studies with a moderate overall score only presented one weak internal component out of the six evaluated [15, 16, 20, 21]. Similarly, those with a weak overall score had only two weak internal components [17, 18].

## 3. Results

### 3.1. Selection of Studies and the Data Extraction Process

After conducting a comprehensive search and applying the controlled terms (DeCS and MeSH) together with Boolean operators as specified in the search strategy, a total of 1306 relevant articles were collected. The first search was conducted in Web of Science (WoS), where 74 articles were found; the second search was conducted in the Scopus database, gathering a sum of 62 articles; the third search was conducted in PubMed, obtaining a total of 10 articles; subsequently, different searches were conducted in ScienceDirect, CINAHL, and LILACS, obtaining a total of 906, 143, and 111 studies, respectively. Eight duplicate articles were eliminated, resulting in a total of 1298 papers.

The criteria detailed in the data extraction were applied. The first level of screening involved examining the titles and abstracts of articles in all databases, retaining those that looked promising for a full-text review. This stage left a pool of 58 articles for further analysis.

Subsequently, inclusion and exclusion criteria were applied to these 58 articles, resulting in the elimination of 48 of them. The reasons for excluding these 48 articles in the context of this review were based on several considerations: insufficient information (*n* = 30) and insufficient discussion of the topic of APN (*n* = 18).

Finally, after this selection process, a total of 10 articles were identified and retained that met the relevance and quality criteria established for this research. To reduce the selection bias, each article was independently reviewed by three of the researchers (C.U.-G., F.-J.G.-V., and E.C.), who decided whether each article met the criteria. If these researchers did not reach a consensus on the inclusion of a paper, the other two researchers (R.C.-B.; M.-d.-l.-A.M.-G. and E.-M.B.-M.) mediated the decision.

### 3.2. Characteristics of the Studies: Result Synthesis

Table 1 provides exhaustive details of the main data related to each of the studies included in this review. These data include relevant information such as names of authors, year of publication, country of origin, type of study, comparisons examined, study objectives, participant population, variables considered, measurement instruments used, interventions, and, finally, the results obtained.

Of the ten research articles included in this review, one (10%) was a longitudinal, multicenter, randomized phase 2 study [15]; one (10%) a cross-sectional study involving two cohorts of patients [16]; three (30%) were qualitative studies [17, 18, 21]; two (20%) were systematic reviews [19, 22]; one (10%) a descriptive cross-sectional study with quantitative approach [20]; one (10%) a quasiexperimental study [23]; and one (10%) a literature review study [24].

In terms of the countries in which the work was carried out, three (30%) were carried out in Switzerland [15, 18, 21], one (10%) in Sweden [16], two (20%) in Spain [17, 20], one (10%) in Brazil [19], one (10%) in Saudi Arabia [22], one (10%) in South Korea [23], and one (10%) in the USA [24].

Concerning the area of study topic addressed by the different articles, five (50%) investigated patients' perceptions of the APN [15, 16, 22–24] two (20%) investigated perceptions of both patients and professionals of the multidisciplinary team [18, 20], two (20%) on the adequacy of APN education [19, 20], and one (10%) on perceptions of patients and family members [21].

Regarding the perceptions of APNs in relation to adult oncology patients, satisfactory results were obtained from patients and other professionals in the multidisciplinary team, with direct clinical practice, coordination, consultation, advice, and education being the most important [17]. In addition, the figure of obtaining information and applying advice on self-management of physical symptoms is positively valued [18]. Similarly, relatives of senior oncology patients also showed their appreciation for this professional, which is considered a valuable resource in the counselling about the disease [21].

From the ten studies analyzed, the following conclusions are drawn regarding the role of advanced practice nurses and the adequacy of their training: They play an essential role in assessing and diagnosing the needs of senior oncology patients by performing a comprehensive geriatric assessment. It also collaborates in the promotion of regular cancer screening and detection [24]; plays a crucial role in the early detection of complications and toxicities related to cancer treatment, contributing to a more accurate diagnosis and more effective care planning [17, 22]; contributes to the assessment and diagnosis of the needs of older patients with colorectal cancer by providing a comprehensive assessment of patients' needs, as well as continuity of care, psychosocial support, and facilitation of self-management of symptoms [18, 23]; and promotes care and facilitates the transition process from diagnosis to end of life. It also plays an important role in education and collaboration with multidisciplinary teams [19]; influences the improvement of patient variables such as uncertainty, ambiguity, inconsistency, and unpredictability, and increases the safety and confidence of patients and relatives [15, 21]; and improves the acquisition of information related to supportive care resources [16]; their training in most cases meets the training standards required by ICN and this is evidenced by their clinical and care practice [19, 20].

### 3.3. Association between the Different APN Interventions with the Quality of Life of Senior Oncology Patients

APNs prioritize and effectively manage pain, providing vital emotional and psychological support during cancer treatment. Through empathetic communication and family participation, they improve both quality of life and patient ability to self-manage their health [19]. In certain cases, the APNs called patients after initial chemotherapy sessions to assess symptoms and provide advice, addressing both physical and psychological aspects of quality of life. This approach was proven to be more effective than interventions that focus solely on targeting quality of life [16]. Emotional and psychological support, with the use of active listening by APNs, also led to improvements in quality of life through reduced stress and anxiety [16]. Another key element was advance care planning in gerontological end-of-life care. People involved in this type of care are more likely to know and fulfill their wishes at the end of life, which improves patient and family satisfaction [24].

The groups with APN counselling showed a better trend of improvement on all uncertainty scales [15]. On the other hand, the reviewed studies have also shown how APNs can provide support to senior oncology patients through external means. The provision of telephone support facilitated access to the system and provided a rapid response to patients' problems and needs [16, 17]. Finally, it was also observed how APNs can make referrals to support services for patients and their families, such as support groups or counselors and mental health services, as they can identify signs of depression or emotional distress and collaborate with other mental health professionals when necessary. All of this had an impact on improving the quality of life of patients and families [15, 19, 24].

## 4. Discussion

The aim of this systematic review was to identify research papers that analyzed the impact of APNs on the quality of life of older adults with cancer.

Cancer care has become increasingly complex due to an ageing population and the growing need for comprehensive and personalized approaches to cancer treatment [1]. This review highlights multiple findings that underscore the essential contribution of APNs in enhancing quality of life and providing comprehensive care to senior oncology patients.

APN collaborates with the multidisciplinary team to carry out a comprehensive geriatric assessment, achieving a substantial change in treatment decisions in more than 40% of patients. With the intervention of this professional, great advances in prevention, detection, and diagnosis are achieved [24]. To grasp the significance of this finding, it is essential to appreciate the role of the global geriatric assessment in adults. Older people undergo a transformation in their health, affecting their functional, psychological, and social aspects. A dependable global geriatric assessment that is valid, feasible, and simple aids in diagnosing health problems across all dimensions. It streamlines medical care and improves the overall quality of life for the elderly [28].

Early implementation of palliative care with APN in cancer treatment significantly improves patient's quality of life, reduces depression, and minimizes the need for intensive care. This approach may also contribute to increased survival, as demonstrated in postoperative patients who received home APN intervention compared to those who did not [15, 19]. These findings emphasize the vital role of APNs in end-of-life care, where their advanced clinical decisions and specialized knowledge in cancer care directly contribute to improved quality of life. This ensures that older adults receive optimal treatment, leading to longer survival in most cases [20].

Nurses are increasingly taking on new roles in different countries. Health education is becoming one of its key functions. There is a significant improvement in patients' perception of the health-related information they receive [16]. All this makes a positive contribution to the patient's experience and to the work of the multidisciplinary team [17].

The health education provided by APNs goes beyond providing general information to senior oncology patients. It plays a crucial role in the ongoing dialogue with patients, offering guidance and education on managing reported side effects during medication treatment [18, 29–31]. Similarly, these nurses work closely with social workers, pharmacists, and physicians. This interdisciplinary and coordinated approach can have a significant impact on the quality of life of senior oncology patients by providing a smoother and more effective transition from the hospital setting to outpatient or home care [16].

On the other hand, it has also been observed that with the intervention of APNs in psychoeducational programs for cancer survivors, their coping improves, as well as their quality of life. This is due to the positive effect on the reduction of stress and anxiety [23]. Patient-centered care, which includes both physical and psychological aspects of quality of life, produces more effective results than interventions that focus solely on quality of life. APNs, with their knowledge and experience, play a crucial role in achieving this, contributing to the reduction of chemotherapy-related symptoms [22, 23]. However, it should be kept in mind that an intervention directed only towards health education is unlikely to lead to a complete behavioural change. To improve the level of behavioural change in senior oncology patients, it is also necessary to implement regular physical activity and dietary practices [32]. Physical activity is an important factor that affects the prognosis and psychosocial adjustment of senior oncology patients [33].

One of the most distressing things for senior oncology patients is the uncertainty they experience. The disease process they are going through has many variables that are completely unknown to them [34]. Raphaelis and collaborators' [15] findings indicate that APN counselling reduces uncertainty related to vulvar neoplasia in adult women. Personalized counselling by APNs showed a significant decrease in uncertainty measures in six months, while written information did not produce significant decreases over time in another group [21]. This corroborates what has already been evidenced in other research, where participants stated that APNs were a key point of support: people who were there for any concerns that were difficult to discuss with a social network or other healthcare providers [35].

In terms of perception of the multidisciplinary team, Serena and collaborators [18] detailed promising results in their research. Physicians perceived APNs as adding value in facilitating access to care, supporting symptom management, providing psychosocial support, and improving continuity of care [18]. These results are consistent with other studies, which emphasize the concurrent empowerment of medicine and nursing. The specialization of nursing roles, such as APNs, contributes significantly to the overarching goal of healthcare, improving patient well-being. Medical professionals recognize and value this nursing role as a valuable complement to their clinical work [36].

Families' perceptions of the APNs were also identified in several studies as favorable [18, 21]. Families noted that, after the intervention, they and their ill relatives received targeted information about cancer, its symptoms, and side effects. In addition, APNs referred them to support services, including self-help groups, sex therapy, and psycho-oncology [18]. However, despite the results, Hart and colleagues [37] estimated in their study that only 65% of patients were willing to be seen by an APN for the first time.

All the scientific literature reviewed agrees that it is necessary to provide individualized, multidimensional and multidisciplinary care to senior oncology patients. For this, the figure of APNs is fundamental, as it is a specialization that requires formal training and education [38, 39]. Currently, there are multiple training initiatives in Spain, such as organizing symposiums, conferences, and courses, although it would still be necessary to include this training and specialization in the curricular training of both geriatric nurses and oncology specialists [9].

This study has limitations, including the heterogeneity of incorporated works due to methodological variations, participant characteristics, intervention specifics, and outcomes. It is difficult to extrapolate conclusions with qualitative studies or systematic reviews; however, in this research, there are also several studies with a control group, a cohort study, and a randomized study, which present greater control of bias [40] and therefore the possibility of extrapolating their conclusions. Despite these limitations, the research addresses a contemporary question in the context of recent global challenges, especially the pandemic. There is a scarcity of studies on the influence of APNs on the quality of life of cancer patient's postpandemic, making this investigation novel. Furthermore, it is a work in which the PRISMA declaration criteria [13] for systematic reviews have been rigorously followed through the exhaustive analysis of the selected articles. However, future research with various interventions and randomized trial designs by APNs is recommended to further advance knowledge in this area, taking into consideration a greater margin of years regarding the publication date for database searches. It would also be appropriate in future research to carry out a meta-analysis after making the systematic review.

## 5. Conclusions

Carrying out systematic reviews such as those in this study is a challenge due to the lack of recognition that currently exists for these professionals. However, the evidence reflected represents relevant conclusions for the profession in particular and for public health in general.

APNs play a crucial role in the care of older adults with cancer, significantly improving their quality of life and contributing to comprehensive patient care. The research conclusions in this review highlight various aspects of the work of APNs in oncological care for the elderly, including comprehensive geriatric assessment, early detection of complications, personalized care planning, emotional support, educational guidance for patients and families, and vital collaboration within multidisciplinary teams. It is evident that advanced practice nurse (APN) is an emerging practice that improves the care of older people.

In summary, the findings presented in this paper endorse the inclusion of APNs in oncology care teams for a better patient experience and overall well-being. It is crucial to acknowledge and appreciate the significant contribution of APNs in oncology. Advocacy for greater recognition and regulation of its role in the care of senior oncology patients, along with its participation in clinical trials, is essential. The work of APNs not only enhances the quality of life of these patients but also positively influences clinical outcomes and the health workforce.

## 6. Implications to Nursing Management

The study highlights the critical role of advanced practice nurses (APNs) in enhancing the care of senior oncology patients, suggesting significant implications for nursing management and leadership. APNs lead multidisciplinary teams effectively, advocating for shared leadership to improve decision-making and care execution. Nursing management should leverage APN expertise in policy advocacy, particularly in expanding their roles within oncology, which is essential for enhancing patient care and operational efficiency.

APNs' ability to manage early complications and provide personalized care underscores the need for ongoing professional development and involvement in quality control measures. Nursing leaders should prioritize continuous education for APNs and empower them to initiate care plan adjustments, ensuring high standards of patient safety and care quality.

Furthermore, establishing robust feedback mechanisms for APNs can enhance the effectiveness of nursing practices. This feedback is crucial for refining patient care strategies and advancing nursing leadership, aligning with the goals of improving clinical outcomes and the overall patient experience in healthcare settings.

## Figures and Tables

**Table 1:** Characteristics of the studies included in the systematic review.

Author (year) country (reference)	Study design	Comparisons	Objectives of the study	Participants	Measured variable and scale	Interventions	Results
Raphaelis, et al. (2018), Switzerland, Austria [15]	Longitudinal, multicentre, randomized phase 2 study	Written information group and counselling group with an advanced practice nurse	To determine whether written information and/or counselling reduces disease-related uncertainty in women with vulvar neoplasia	*N* = 49 women with vulvar neoplasia from four Swiss hospitals and one Austrian hospital	The German adult form of the Mishel uncertainty of illness scale (MUIS-A)	Written: two booklets during the time from diagnosis to surgery. Counselling: five consultations with APN for 10–50 minutes	Overall, the counselling group showed a better trend of improvement on all uncertainty scales throughout the study. Within the counselling group, uncertainty, ambiguity, inconsistency, and unpredictability decreased significantly over six months

Westman et al. (2019), Sweden [16]	Cross-sectional study including two cohorts of patients	All patients with gynecological, hematological, head and neck CA and upper GTI before and after the intervention of a new nursing figure	To compare patients' perceptions of care before and after the introduction of a new advanced nursing role, the coordinating contact nurse (CCN), in a region of Sweden	*N* = 1872 [patients with gynecological (*n* = 598), hematological (*n* = 461), upper gastrointestinal (*n* = 418) and head and neck cancers (*n* = 395)]	The validated Swedish versions of the European Organization for Research and Treatment of Cancer (EORTC) quality of life questionnaire, QLQ-C30 and QLQ-INFO25, and also a study-specific questionnaire	Interventions were carried out by the new nursing staff. Data were collected in April-May 2015 (baseline) and April-May 2017 (follow-up)	In relation to health-related patient information (overall mean EORTC QLQ- INFO25 score increased from 41.23 to 44.16, *p*=0.0006), statistically significant improvements were found related to the availability of supportive care resources, e.g., increased informed access to the contact nurse and individual written care plans
Serra-barril et al. (2023), Spain [17]	Qualitative phenomenological study	A group of patients and a multidisciplinary group of professionals	To know the lived experience of cancer patients and multidisciplinary professionals in relation to the care provided by the advanced practice nurse	18 professionals and 11 patients	Experiences related to advanced practice nurse, most relevant functions/characteristics/care offered by this figure, benefits of the care provided, and most relevant aspects of care. The instrument used was self-developed interviews	The study took place from March to December 2021 in four highly complex public university hospitals in Catalonia. Individual interviews with professionals (45–60 minutes each) were conducted online from March to May 2021, using the Microsoft teams platform. From October to December 2021, patient interviews were conducted in person, in a quiet and comfortable hospital room, and lasted approximately 30–45 min	Advanced practice nurses play a critical role in cancer care, making positive contributions to the patient experience and to the work of the multidisciplinary team

Serena et al. (2018), Switzerland [18]	Qualitative descriptive	A multidisciplinary group of professionals and a group of patients	To explore the acceptance of a new role, the advanced practice lung cancer nurse (APNLC), from the perspective of patients and healthcare professionals in a country that lacks regulatory oversight of advanced practice nurse (APN) roles	Multidisciplinary healthcare team, including physicians (*n* = 6), oncology nurses (*n* = 5), the social worker, and the APNLC. Patients (*n* = 4)	The measurement variables were identification of the role of the CLNPA, specific contributions of the role of the CLNPA, and flexibility of the CLNPA service. Semistructured interviews with self-developed items and a self-developed guide for conducting focus group discussions were used for this purpose	Two focus group discussions were conducted with members who had worked closely during the last 6 months with the ANPLC: G1 nurses and social workers and G2 physicians. Semistructured interviews were conducted for ANPLC and patients (lung cancer patients who had received care from ANPLC)	Three main themes were found: identification of the role of the CLNPA, specific contributions of the role of the CLNPA, and flexible service of the CLNPA. Clinicians and patients clearly recognized the role of the CLNPA, noting contributions to continuity of care, psychosocial support, and facilitation of self-management of symptoms
Schneider, Kempfer, and Backes (2021) Brazil [19]	Systematic review	—	Seeking evidence on the education of advanced practice nurses through clinical practice and nursing care with cancer patients	A total of 12 experimental studies were identified	The variables that the studies had to address were educational guidance, control of pain or other symptoms related to the disease and/or treatment, and satisfaction and improvement in the quality of life of cancer patients. All of them in studies in which advanced practice nurses had been involved	The searches were carried out in the following electronic data: PubMed, LILACS, Institute for Scientific Information (ISI) Web of Knowledge via Web of Science, Scopus, CINAHL-EBSCO, and Cochrane Central Register of Controlled Trials (CENTRAL). The timeframe was from 15 July 2018 to 15 December 2019	The analysis of these studies showed that advanced practice nurses were adequately trained. This fact was objectified through the conclusions of the experimental studies analyzed, since, through the good practice of advanced practice nurses, an improvement in the control of pain or other symptoms related to the disease and/or the treatment, satisfaction, and improvement in the quality of life of cancer patients was identified

Muñoz et al. (2023), Spain [20]	Cross-sectional, descriptive study with a quantitative approach	Intervention without a control group	To identify the competency profile of advanced practice nurses involved in the care process of oncology patients	*N* = 35 nurses caring for cancer patients at the Hospital del Mar, Barcelona	The variables analyzed were the educational standards required by ICN for advanced practice nurse and postgraduate academic qualifications. The instrument for defining the role of the advanced practice nurse (IDREPA) was used	The data were recorded between February and March 2021 through the completion of the instrument by the professionals	Nine (31%) nurses were identified as meeting the standard in all 6 domains on the IDREPA scale to be considered as advanced practice nurses. Of these 9, 31% met the educational standards required by ICN, 7 (24.1%) with an official master's degree and 2 (6.9%) with a doctorate
Geese et al. (2020), Switzerland [21]	Qualitative analysis study	A single experimental group with two subgroups, one of the patients and one of the relatives of patients	To explore the experience of prostate cancer patients undergoing radical prostatectomy and their partners from diagnosis through to follow-up care and the APN support program	*N* = 18 participants, patients (*n* = 10) spouses (*n* = 8)	The study variables were perceptions of empathetic, trusting, informed, open behaviours, and quality of information received in terms of diagnoses and symptoms, offered by the advanced practice nurse. A series of self-developed semistructured interviews were used as an instrument	Between September 2015 and January 2016, 10 patients with PCa and eight spouses agreed to participate in the study. A series of semistructured interviews were conducted to explore patients' and their partners' experiences from diagnosis to discharge	Patients appreciated the EPA's support program. They noted that the EPA was empathetic, trustworthy, knowledgeable, and open. Patients received specific information about PCa, including related symptoms and postoperative side effects

Alotaibi and Al anizi. (2020), Saudi Arabia [22]	Systematic review	—	To determine how advanced practice nurses (APNs) can contribute to cancer care	*N* = 5 items that met the established criteria	The variables taken into account in this work were support for elderly patients, stress relief, improvement of quality of life, and help in symptom management	A series of systematic searches of research studies conducted from 2005 to 2018 were carried out in the following databases: MEDLINE, CINAHL, PubMed, and AMED	The selected studies showed that EPAs provide support to elderly patients, which helps to relieve stress and improve the quality of life of cancer patients. In addition, it was found that EPAs can help patients with symptom management
Kim and Yoo (2022), South Korea [23]	Quasiexperimental study with a pretest-posttest nonequivalent control group	Experimental and control groups	To investigate the effects of an advanced practice nurse-led psychoeducational program on distress, anxiety, depression, cancer coping, health-promoting behaviour, and quality of life among colorectal cancer survivors	*N* = 39. 19 in the experimental group and 20 in the control group	Distress, anxiety, depression, cancer coping, health promotion, and QOL (quality of life) were investigated	A psychoeducational program was implemented by an advanced practice nurse. The program included interventions on anxiety, depression, distress, coping and health-promoting behaviours, and quality of life. Variables were measured before, immediately after, and 4 weeks after the intervention	The psychoeducation program had a positive effect on reducing stress and anxiety in colorectal cancer survivors, improving their coping with cancer and their quality of life

Morgan et al. (2016), USA [24]	Bibliographic review article	—	Describe how the advanced practice nurse (APN) is uniquely suited to meet the needs of older adults across the cancer continuum	*N* = 82. The information detailed in this paper is based on 82 articles found in searchable databases	Cancer care through: (1) preventive care, screening, and early diagnosis; (2) oncology and gerontology-specific care in geriatric oncology clinics and beyond; and (3) throughout survivorship	A series of searches were carried out in the following databases: Google Scholar, PubMed, and CINAHL. Search terms included the following: “Gero-oncology,” “geriatric oncology,” “advanced practice nurse,” “nurse practitioner,” “older adult,” “elderly,” and “cancer”. The papers included in this review range from 2002 to 2015	APNs have made great strides in the care of older adults with cancer through prevention, screening and diagnosis, evidence-based geriatric oncology, and throughout the disease process and are well positioned to help understand the complex relationship between risk factors, geriatric syndromes, and frailty and translate research into practice

**Table 2:** Quality assessment components and ratings for the EPHPP instrument.

Articles	Components^∗∗^						Overall score^∗^
	1	2	3	4	5	6	
Raphaelis et al. [15]	S	S	M	W	S	S	M
Westman et al. [16]	S	M	M	M	S	S	M
Serra-barril et al. [17]	M	W	S	W	M	S	W
Alotaibi and Al [22]	M	W	M	W	M	S	W
Muñoz et al. [20]	S	W	S	M	S	S	M
Geese et al. [21]	M	M	M	M	W	S	M
Kim et al. [23]	S	S	M	M	S	S	S

^∗^W, weak; M, moderate; S, strong. ^∗∗^1 = risk of bias; 2 = design; 3 = confounding factors; 4 = masking; 5 = data collection; 6 = withdrawals.

## Data Availability

The data used to support the findings of this study are included within the article.
